# The dual role of the Notch signaling pathway in digestive system cancers

**DOI:** 10.1186/s40001-026-03947-3

**Published:** 2026-01-27

**Authors:** Dairong Xiang, Tuokai Wang, Shihui Wei, Ruihong Zhao, Lanjuan Li, Juan Lu

**Affiliations:** 1 State Key Laboratory for Diagnosis and Treatment of Infectious Diseases, National Clinical Research Center for Infectious Diseases, China-Singapore Belt and Road Joint Laboratory on Infection Research and Drug Development, National Medical Center for Infectious Diseases, Collaborative Innovation Center for Diagnosis and Treatment of Infectious Diseases, The First Affiliated Hospital, Zhejiang University School of Medicine, Hangzhou, Zhejiang China; 2 Yuhang Institute of Medical Science Innovation and Transformation, Hangzhou, Zhejiang China

**Keywords:** Notch signaling, Digestive system cancers, Dual role, Tumor microenvironment, Targeted therapy

## Abstract

The Notch signaling pathway is critical for maintaining tissue homeostasis and plays dual roles in digestive system cancers, acting both as an oncogene and a tumor suppressor gene. This article explores its varied functions across esophageal, gastric, liver, pancreatic, and colorectal cancers. In esophageal and pancreatic cancers, Notch signaling may initially inhibit tumor growth but later promote progression, influenced by the primary cell types. In hepatocellular carcinoma, DLL4/Notch1 generally drives tumor growth, whereas Jag1/Notch2 tends to suppress tumor progression. In colon cancer, this pathway not only facilitates immune evasion but, in the presence of specific mutations, can also enhance the anti-tumor immune response. The functional complexity of Notch signaling presents significant therapeutic challenges, as broad-spectrum γ-secretase inhibitors (GSIs) are often associated with considerable side effects. Future treatment strategies should prioritize precision medicine, including subtype-specific Notch receptor inhibitors, biomarker-driven personalized therapies, and combination treatments aimed at modifying the tumor microenvironment. A thorough understanding of these dual roles is significant for developing more accurate and effective treatment approaches for digestive system cancers.

## Introduction

The global burden of digestive system cancers remains substantial. In 2021, malignancies of the digestive system accounted for 39.29% of cancer-related deaths, making them the leading cause of cancer mortality. This category includes gastric, colorectal, and esophageal cancers. The age-standardized mortality rates (ASDR) for colorectal and gastric cancers were 12.40 and 11.20 per 100,000 people, respectively, ranking among the top four causes of cancer death [1, 2]. Significant differences in age, gender, socio-economic status, dietary habits, and geographical distribution pose challenges for personalized treatment strategies. Given the limitations of current diagnostic technologies and therapeutic agents, investigating the mechanisms of carcinogenesis and progression, as well as identifying new therapeutic targets, is essential for improving patient survival rates [3–7].

As research advances, treatment strategies have evolved from standardized approaches to precision medicine and immunotherapy [8, 9]. The Notch signaling pathway is a highly conserved mechanism across multicellular organisms [10–16]. First identified in 1917 through the study of Drosophila mutants, its molecular characterization was completed in 1983 [17, 18]. The Notch signaling cascade plays a pivotal regulatory role in organismal development, governing key biological processes such as organogenesis, tissue homeostasis, and regeneration. Disruption of this pathway often leads to pathological states, particularly through its oncogenic potential in tumorigenesis [19, 20].

Recent studies have highlighted the regulatory role of Notch signaling in various carcinogenic processes, including cell proliferation, metastatic migration, immune microenvironment regulation, and epithelial–mesenchymal transition (EMT) [21–25]. Notch exerts both oncogenic and tumor-suppressive effects. Its cancer-promoting function was first identified in leukemia and later confirmed in skin cancer and lung squamous cell carcinoma (LUSC) [26, 27]. Studies on melanoma cells have also demonstrated their anti-cancer properties [28–30]. This review synthesizes current knowledge on the dual regulatory mechanisms and modulating factors of the Notch signaling pathway in digestive system cancers and explores potential therapeutic strategies leveraging this duality for targeted interventions.

### Core molecular mechanism of the Notch signaling pathway

**Fig. 1:**
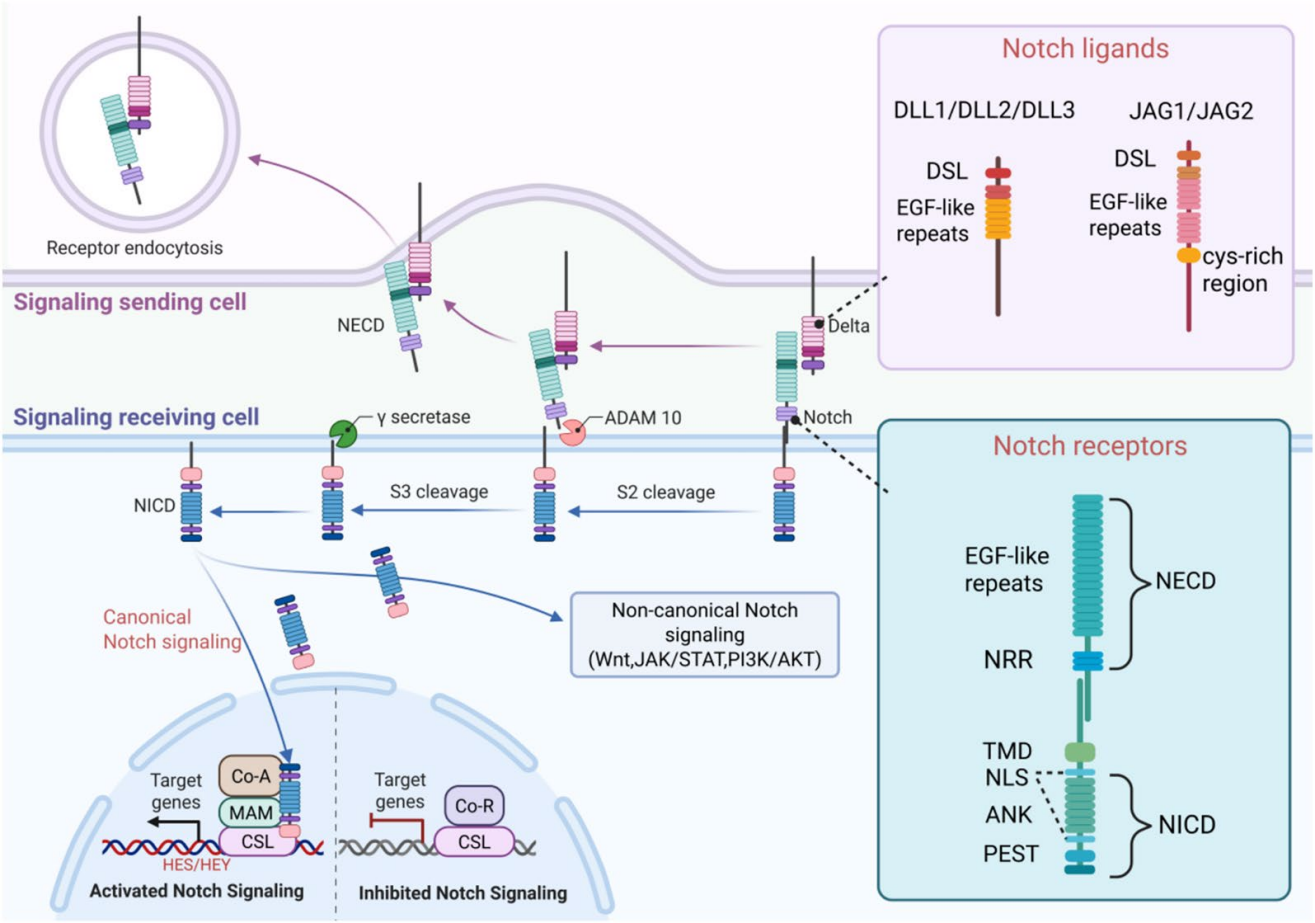
Notch signaling pathway: composition, structure, and activation. The Notch signaling pathway mediates intercellular communication through four transmembrane receptors (Notch1–4) and five corresponding ligands (Jag1, Jag2, DLL1, DLL3, and DLL4). Each ligand possesses a unique molecular structure. The binding of a ligand to receptors on the surface of adjacent cells initiates signal transduction, a highly regulated process. This interaction induces a conformational change in the receptor, exposing its extracellular domain, which is subsequently cleaved by the ADAM protease. (Created by biorender.com)

The Notch receptor family consists of four structurally similar members: Notch1, Notch2, Notch3, and Notch4 (Fig. 1). Each subtype contains three fundamental domains: the extracellular domain (NECD), the transmembrane domain (NTM), and the intracellular domain (NICD) [31]. Together, these domains form the core structure of the Notch signaling pathway. The NECD region is characterized by multiple epidermal growth factor (EGF)-like repeat units and a negative regulatory region (NRR). Post-translational modification through O-glycosylation plays a pivotal role in ligand binding specificity, contributing to the intricate regulatory mechanisms of Notch signaling [32]. Specifically, the NECD of Notch1–4 contains 36, 36, 34, and 29 EGF-like repeats, respectively, which are critical for receptor–ligand binding [33]. The NRR contains three cysteine-rich Lin12/Notch repeat modules that link the NECD with the membrane-associated NICD, maintaining the structural integrity required for receptor processing [34–36]. In gastrointestinal cancers, the functional roles of different Notch receptor subtypes vary [37]. Notch1 and Notch2 typically promote tumorigenesis, whereas Notch3 and Notch4 are thought to inhibit tumor growth. These functional discrepancies are primarily driven by differences in transcriptional regulation mediated by their respective intracellular domains [38]. This fine regulatory mechanism likely underlies the "double-edged sword" effect observed in Notch signaling.

Both Notch receptors and their ligands are transmembrane proteins in humans. The ligand family includes three Delta-like proteins—DLL1, DLL3, and DLL4—and two Jagged proteins: Jag1 and Jag2 [39, 40]. Structurally, the extracellular portions of Jag1 and Jag2 contain a DSL domain (Delta, Serrate, and LAG-2), multiple EGF-like repeats, and a cysteine-rich region. In contrast, DLL1, DLL3, and DLL4 share similar extracellular domains but lack the cysteine-rich segment [41]. Downstream signaling is mediated by intracellular effector complexes, such as γ-secretase and the nuclear CSL complex, which transmit signals to the nucleus and activate the transcription of target genes [21, 33, 42]. The Notch pathway facilitates direct intercellular communication through ligand–receptor interactions. When ligands on signaling cells bind to receptors on adjacent cells, they trigger ADAM protease-mediated cleavage (via ADAM10 or ADAM17) of the Notch extracellular domain. This is followed by γ-secretase-dependent hydrolysis of the intracellular domain, generating the NICD [43–46]. The NICD then regulates various physiological processes through the canonical Notch signaling pathway [39].

In the canonical Notch signaling pathway, this process leads to the release of the soluble NICD from Notch. The NICD then translocates to the nucleus, where its RAM domain interacts with the transcription factor CBF1/suppressor of hairless/Lag1 (CSL, also known as RBPJ) [47]. This interaction activates the transcription of genes within the HES/HEY family. Utilizing their basic helix-loop-helix (bHLH) domains, these proteins bind to DNA and act as repressors of genes involved in cellular differentiation [48]. Emerging evidence highlights the importance of the Notch-HES6 pathway in cancer, particularly in uveal melanoma, where HES6 promotes oncogenic progression through transcriptional inhibition of PTEN [49, 50]. Furthermore, HEY family proteins are involved in a feedback loop with SOX2, modulating the strength of Notch signaling by binding directly to RBPJ [51].

### Dual role of the Notch pathway in esophageal cancer tissue-specific expression profiles of Notch receptor subtypes

In esophageal squamous cell carcinoma (ESCC), aberrant activation of the Notch signaling pathway is frequently observed, with various receptor subtypes exhibiting distinct tissue-specific expression profiles. Studies show that clones harboring Notch1 mutations are often present in normal esophageal epithelium but occur less frequently in tumor tissues. This suggests that such mutations may facilitate clonal expansion while inhibiting tumor progression [52]. These biallelic mutations impair Notch1-mediated signal transduction, leading to distinct gene expression patterns in aging human esophageal samples [52]. Moreover, dysregulation within the UBE3A-ZNF185/Notch signaling axis has been identified as a critical factor in the progression of ESCC [53].

### Tumor-promoting mechanisms in esophageal cancer

**Fig. 2:**
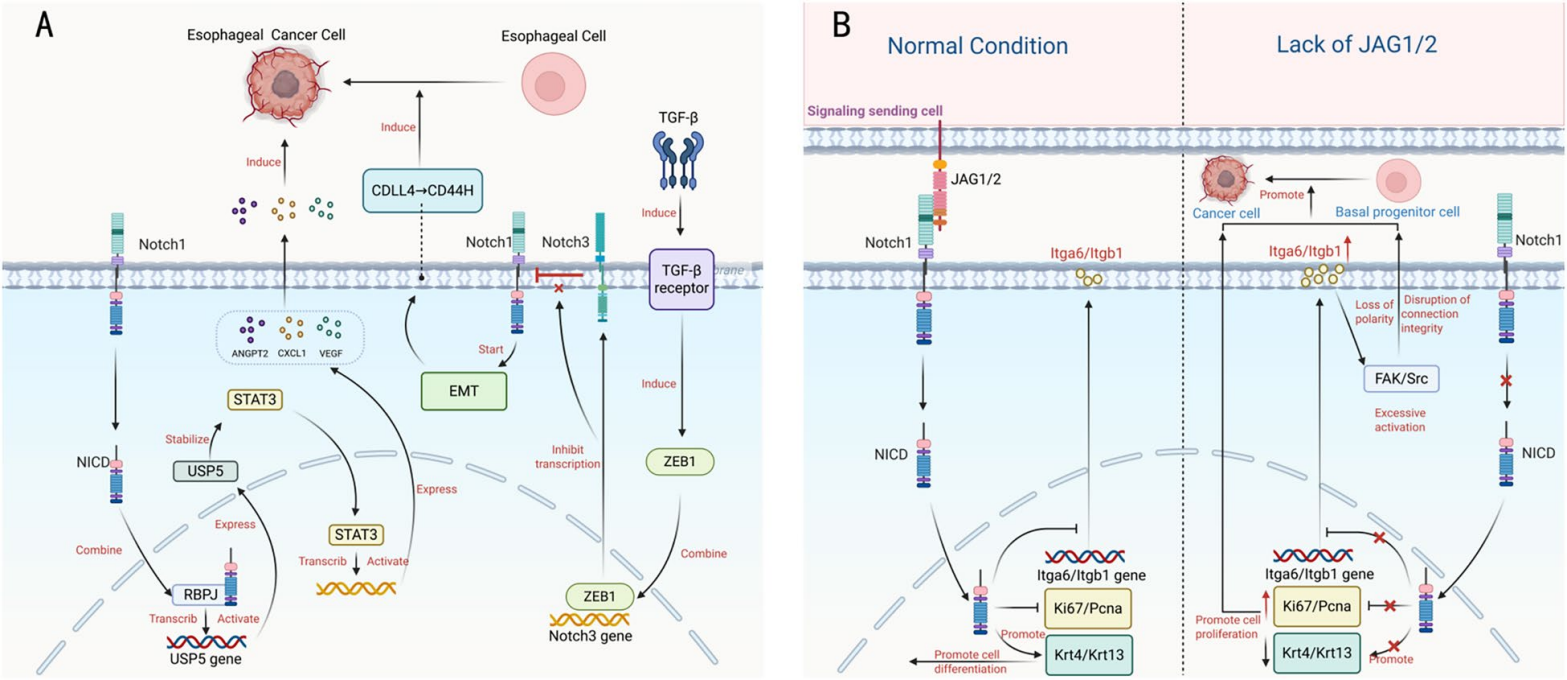
The dual role of Notch signaling in esophageal cancer. **A** Notch signaling promotes esophageal cancer progression via two key mechanisms. The Notch1–USP5–STAT3 axis drives angiogenesis and proliferation by stabilizing STAT3, which increases the secretion of pro-angiogenic factors. Meanwhile, the TGFβ–ZEB1 pathway suppresses Notch3, inducing epithelial–mesenchymal transition (EMT) and generating tumor-initiating cells. **B**. Mechanism of Jag1/2-mediated Notch signaling in maintaining esophageal epithelial homeostasis and suppressing tumorigenesis. Under normal conditions, Jag1/2 activates Notch signaling to maintain tissue homeostasis by balancing cell proliferation and differentiation. In contrast, Jag1/2 deficiency impairs Notch signaling, leading to uncontrolled proliferation, defective differentiation, disruption of tissue architecture, and ultimately driving tumorigenesis. (Created by biorender.com)

Acquiring mesenchymal traits promotes malignant transformation by inhibiting senescence induced by oncogenes, a phenomenon frequently observed through elevated Twist1/Twist2 expression in various human cancers. These proteins inhibit key regulatory factors in p53 and RB-dependent signaling pathways, thus enabling cells to avoid premature aging triggered by oncogenes [54]. Similarly, overexpression of EGFR can induce oncogene-induced senescence; however, certain cells evade this by upregulating ZEB transcription factors (ZEB1 and ZEB2), which, along with EMT induced by TGF-β, allow for continued proliferation (Fig. 2. A) [55]. Further studies have demonstrated that the Notch1 and TGFβ–ZEB1 axis acts synergistically in squamous cell carcinoma (SCC). Mechanistically, TGF-β stimulates the binding of ZEB1 to Notch3 introns, leading to transcriptional inhibition, which promotes EMT and the conversion of tumor-initiating cells from CD44L to CD44H expression (Table 1) [56]. These findings clarify the role of Notch1 in tumor progression in SCC and provide new insights into cancer heterogeneity and treatment resistance. The TGFβ–ZEB1–Notch1 pathway has emerged as a potential therapeutic target, paving the way for precision medicine in SCC.

**Table 1:** Direct regulation of digestive system cancers by the Notch pathway

Tumor type	Notch member	Function (oncogenic/tumor-suppressive)	Pathway crosstalk	Downstream core mechanism and effects	Molecular marker/target	Ref
ESCC	Notch1	Oncogenic	TGF-β	Synergizes with the TGFβ-ZEB1 axis to suppress Notch3, driving EMT	ZEB1, Notch3	[46]
ESCC	Notch1	Oncogenic	STAT3	Activates USP5 transcription to stabilize STAT3, promoting angiogenesis (via VEGF)	USP5 (inhibitor target)	[53]
ESCC	Notch1 (mutant)	Tumor-suppressive	–	Loss-of-function mutations induce cell cycle arrest and differentiation, inhibiting carcinogenesis	Notch1 mutation status	[42, 54, 58]
ESCC	(Jag1/2)/Notch1	Tumor-suppressive	–	Maintains basal progenitor cell polarity and homeostasis; loss accelerates carcinogenesis	Jag1/2 expression	[56]
GC	Notch1/2	Oncogenic	–	CARM1-mediated methylation of N2ICD enhances its activity, promoting proliferation and EMT	CARM1	[63–65]
GC	Notch3	Oncogenic	Akt-mTOR	Upregulated via miR-491/875-5p suppression; drives progression by activating the Akt–mTOR pathway	miR-491/875-5p	[66, 67]
HCC	DLL4/Notch1	Oncogenic	c-myc	Promotes proliferation (via Cdk1 upregulation) and metastasis (via the c-myc–VCAM1 axis)	DLL4, VCAM1	[78, 79]
HCC	Jag1/Notch2	Tumor-suppressive	–	Inhibits proliferation (via p21 induction) and antagonizes Notch1 signaling by suppressing DLL4	p21	[79–81]
HCC	Notch3	Oncogenic	E2F2	Activated by the SNORA74A–DCAF13–E2F2 axis to promote liver CSC self-renewal	SNORA74A	[83]
PDAC	Jag1/Notch	Bidirectional	–	Early-stage tumor suppression: Inhibits Kras-driven cystic tumor formation Late-stage oncogenic: see Table 2 for the mechanism involving macrophage crosstalk	Tumor developmental stage	[87, 88]
PDAC	Notch1	Bidirectional	–	Oncogenic (acinar-derived): inhibits differentiation Tumor-suppressive (embryonic-derived): restrains progenitor proliferation	Cell of origin	[89–94]
PNET	Notch	Bidirectional	p53	Oncogenic (p53-WT): promotes proliferation via INSM1 nuclear translocation Tumor-suppressive: expression is lost in malignant PNETs	INSM1, p53 status	[95–97]
CRC	Notch1	Oncogenic	–	Overexpression correlates with advanced pathological features (invasion, metastasis)	Notch1 expression	[99]
CRC	Notch pathway	Tumor-suppressive	–	Loss-of-function relieves inhibition of the cell cycle regulator p27, causing hyperproliferation	p27	[107, 108]

By maintaining cancer stem-like properties in tumor cells, the Notch signaling pathway significantly enhances the invasiveness of esophageal carcinoma [57, 58]. Dysregulated Notch activity promotes tumor development and progression in esophageal cancer through its role in facilitating EMT [59]. Research by Zheng et al. showed that elevated UBE3A expression in esophageal cancer accelerates disease progression through ubiquitin-mediated degradation of ZNF185, which subsequently removes inhibitory regulation on the Notch signaling cascade. Their work established the UBE3A–ZNF185–Notch axis as a key regulatory pathway in esophageal cancer progression [53]. Additionally, Notch signaling coordinates interactions between malignant cells and other components of the tumor microenvironment (TME), supporting the maintenance of cellular "stemness" and contributing to the specification of the cancer stem cell (CSC) niche [60].

Ubiquitin-specific protease 5 (USP5) exerts significant tumor-promoting effects in both KRAS-mutated pulmonary malignancies and hepatocellular carcinoma (HCC) [61, 62]. Recent studies by Li et al. have shown that in ESCC, USP5 functions as an effector enzyme in the Notch1–USP5–STAT3 axis, promoting tumor angiogenesis and enhancing cancer cell proliferation. This pathway activates USP5 transcription through the NICD1–RBPJ complex, and USP5 stabilizes STAT3 via deubiquitination, leading to the secretion of angiogenic factors such as VEGF, ANGPT2, and CXCL1, thereby inducing tumor angiogenesis [63]. Furthermore, experimental evidence indicates that inhibiting Notch signaling effectively restricts vascular formation in ESCC, as demonstrated in cellular models and animal models. NICD1 overexpression can enhance this inhibitory effect. In light of the previously described mechanisms of USP5 in tumorigenesis, researchers found that the USP5 inhibitor EOAI3402143 attenuates angiogenesis and tumor growth in NICD1-overexpressing ESCC, with combination chemotherapy showing enhanced therapeutic outcomes [63]. The elucidation of the Notch1–USP5–STAT3 axis in promoting tumor progression and the development of related inhibitors offers novel targets for anti-angiogenic therapy in ESCC.

### Tumor-suppressive mechanisms in esophageal cancer

The Notch signaling pathway plays a complex dual role in esophageal cancer, functioning both as a tumor promoter and a tumor suppressor. The differential expression of Notch1 mutations in normal esophageal epithelium versus tumor tissues suggests a potential tumor-suppressive function of Notch1 [52, 64]. Basal precursor cells are crucial for maintaining the homeostasis of the esophagus and anterior stomach, and their dysfunction can promote inflammation and tumorigenesis [65]. Jag1/2 plays a central role in preserving the structural integrity of the esophageal and anterior gastric epithelium (Fig. 2. B). Deletion of Jag1/2 directly disrupts epithelial structure, leading to basal cell proliferation, expanded cell gaps, and thinning of epithelial layers. Jag1/2 regulates basal progenitor cell homeostasis through the Notch signaling pathway, and its deletion reduces Notch1 activity, upregulating proliferation markers (Ki67 and PCNA) while downregulating differentiation markers (Krt4 and Krt13). This disrupts the dynamic balance between progenitor cell proliferation and differentiation. Notably, Jag1/2 deletion also increases the expression of basal cell adhesion molecules (Itga6 and Itgb1), impairing cell polarity and junctional integrity, which exacerbates epithelial dysfunction. Under pathological conditions, the effects of Jag1/2 deletion become even more pronounced: it not only worsens DCA-induced anterior gastric epithelial injury (leading to more severe inflammation, basal cell proliferation, and dysplasia) but also accelerates the development of 4-NQO-induced anterior gastric SCC. This process is accompanied by increased immune cell infiltration in tumor tissues and a decreased survival rate in mice. This study concludes that in human ESCC, the dynamic expression of Jag1/2 is closely linked to the progression of carcinogenesis. Its expression decreases in the early stages of inflammation and dysplasia but increases significantly when ESCC is diagnosed [66]. Collectively, these findings highlight the critical role of Jag1/2 in esophageal homeostasis, its regulation of basal progenitor cell polarity and junctional integrity via Notch signaling, and its tumor-suppressive role during ESCC initiation, offering potential biomarkers for early diagnosis.

In high-grade tumors, Notch signaling suppresses neuroendocrine differentiation and modulates tumor cell lineage plasticity [67]. Moreover, the Notch pathway can induce cell cycle arrest and promote differentiation in esophageal cancer cells by regulating the expression of cell cycle-related proteins [68].

### Impact of microenvironmental factors on functional polarization

The TME of esophageal carcinoma plays a critical role in modulating the functional output of Notch signaling [67]. The combined use of radiotherapy and anti-angiogenic therapy can mitigate the immunosuppressive state within the esophageal cancer TME. Tumor-associated macrophages (TAMs), the most prevalent immune cell type in the TME, exhibit elevated expression of Notch receptors [69, 70]. Activation of this signaling pathway promotes the production of immunosuppressive mediators [71]. Chemoradiotherapy (CRT) reshapes the esophageal cancer TME by upregulating tumor-specific antigens and immune checkpoint molecules, inducing both immunogenic and immunosuppressive responses [72]. Additionally, the NRF2/Notch interaction contributes to metabolic reprogramming, further remodeling the TME to support malignant tumor progression [73].

### Regulatory characteristics of the Notch pathway in gastric cancer

#### Interaction between *Helicobacter pylori* infection and Notch signaling

Infection with *Helicobacter* *pylori*, a primary contributor to gastric cancer development, disrupts the homeostatic balance of the gastric epithelium by modulating Notch pathway activity. Research shows that Notch1 and Notch2 receptors play critical roles in regulating gastric stem cell behavior. The ligand DLL1, essential for Notch signaling, controls cellular proliferation in the antral region of the stomach. Cells expressing DLL1, located at the bases of glands, function as specialized niche components that activate adjacent LGR5-positive stem cells through paracrine signaling, thereby maintaining stem cell expansion and epithelial homeostasis [74]. In the H. pylori-infected microenvironment, interactions between these receptors and specific ligands become dysregulated, resulting in aberrant stem cell proliferation. Moreover, the disruption of Notch signaling’s role in maintaining the balance of gastric epithelial differentiation may facilitate the metaplasia–dysplasia–carcinoma sequence [21, 74].

#### Mechanisms of Notch1/2 in gastric cancer

Notch1 and Notch2 exhibit significant carcinogenic properties in gastric cancer. These receptors promote cancer cell invasiveness by preserving tumor stem cell characteristics and contributing to the EMT, thereby facilitating gastric cancer metastasis [21, 73, 75, 76]. Mechanistically, activation of Notch1/2 induces metabolic reprogramming, alters immune cell function within the TME, and creates an immunosuppressive environment conducive to tumor progression [53]. Additionally, in gastric cancer, Nup54 promotes the nuclear transport of CARM1 through physical interactions, establishing the foundation for nuclear regulation. Nuclear CARM1 regulates the Notch2 pathway via two mechanisms: first, it binds to the Notch2 promoter along with TFEB, activating Notch2 transcription through catalytic histone H3R17 methylation. CARM1 serves as a key cofactor in TFEB-mediated transcription activation. Second, the EVH1 domain methylates residues R1786, R1838, and R2047 in the AR domain by interacting with the pest domain of the active Notch2 intracellular domain (N2ICD). This methylation enhances N2ICD’s interaction with the coactivator MAML1, thus activating downstream Notch2 target genes and promoting gastric cancer cell proliferation, colony formation, and in vivo tumorigenesis. This defines a complete regulatory axis: "CARM1 → Notch2 pathway activation → maintenance of malignant phenotypes in gastric cancer" [77]. These mechanisms highlight the tumor-promoting roles of Notch1 and Notch2 signaling pathways.

#### Evidence for the role of Notch 3/4 in gastric cancer

Unlike Notch1 and Notch2, Notch3 exhibits a distinctive dual functionality in gastric cancer. Elevated expression levels of Notch3 have been consistently observed in gastric tumor specimens [78, 79]. This regulation is mediated by the suppression of miR-491-5p and miR-875-5p, through which Notch3 functions as a transcriptional co-activator that directly modulates PHLDB2, thereby activating the Akt–mTOR signaling cascade and facilitating the initiation and progression of gastric malignancies [80]. Notably, both Notch3 and Jagged2 expressions are not only correlated with tumor development but also positively associated with improved histological differentiation of gastric cancer toward intestinal/ductal subtypes, suggesting their potential as favorable prognostic markers [81]. Additionally, Notch4 has demonstrated tumor-inhibitory activity. Clinical analysis revealed that patients with Notch4 mutations in the discovery cohort showed significantly improved objective remission rates (42.9% vs. 25.9%) and disease control benefits (54.0% vs. 38.1%). These patients also exhibited significantly prolonged progression-free survival (HR = 0.558) and overall survival (HR = 0.568). In the independent validation cohort of 1423 patients, those with Notch4 mutations had a significantly longer median overall survival (41.0 months vs. 18.0 months). However, no survival difference was observed in the subgroup not receiving immune checkpoint inhibitor (ICI) treatment. Mechanistically, Notch4 mutation is linked to an increased tumor mutation burden (TMB), a higher ratio of non-synonymous to synonymous mutations, and an elevated single-nucleotide polymorphism/insertion-deletion antigen load, all indicating enhanced immunogenicity. Tumors harboring these mutations also exhibited upregulated expression of MHC molecules, co-stimulatory factors, PD-1, PD-L1, CTLA4, and other immune checkpoint proteins, along with increased infiltration of CD8^+^ T cells and tumor-infiltrating lymphocytes. These features were associated with greater diversity in T cell receptors, enhanced cytolytic activity, and increased production of immunostimulatory chemokines such as CXCL9 and CXCL10, collectively reflecting enhanced anti-tumor immune activation [82–84]. These findings demonstrate the critical role of Notch3/4 in gastric cancer, highlighting new therapeutic opportunities for targeting these molecular pathways.

#### Tumor heterogeneity and receptor expression variation

Molecular studies have identified several interconnected signaling pathways, including Wnt, hedgehog, and Notch, as pivotal in the progression, treatment response, and metastasis of gastrointestinal cancer [21, 42]. Gastric cancer exhibits considerable tumor heterogeneity, evident from the significant variation in Notch receptor expression across different patients [85]. Early research has shown that the heterogeneous expression profiles of Notch1 through Notch4 receptors can even vary within different regions of the same tumor [86]. This variability is partly driven by TME factors, particularly the interaction between the Notch pathway and other signaling networks, such as PI3K/AKT/mTOR [21, 85, 87]. Notably, differences in the intensity of Notch signaling can produce opposing effects of the same receptor in distinct gastric cancer subtypes (Table 2) [88], complicating the design of targeted therapies [89]. Given these complexities, exploring combined treatment strategies targeting Notch signaling, alongside continued preclinical and early clinical research, holds promise for advancing from mechanistic understanding to precise cancer treatment.

**Table 2:** Indirect regulation by the Notch pathway via the microenvironment and stem cells

Regulatory dimension	Tumor type	Notch member/pathway	Function(oncogenic/tumor-suppressive)	Regulated target	Pathway crosstalk	Core regulatory mechanism and final effect	Molecular marker/target	Ref
Immune regulation	GC	Notch4 (mutant)	Tumor-suppressive	T cells, NK cells	JAK–STAT	Mutations increase TMB/NAL and upregulate MHC/PD-L1, boosting CD8^+^ T cell and NK cell infiltration and function	Notch4 mutation status	[68–72]
	CRC	Notch4	Oncogenic	TAMs	STAT1/STAT3	Promotes an immunosuppressive TAM phenotype by suppressing STAT1 and enhancing STAT3 activity	Notch4 in TAMs	[98]
	CRC	Notch3	Oncogenic	Macrophages, MDSCs	AKT	Upregulates CSF1/CXCL12 to recruit immunosuppressive cells, promoting liver metastasis	CSF1, CXCL12	[100, 101]
	CRC	Notch2	Tumor-suppressive	cDC1 dendritic cells	–	Enhances cDC1 differentiation, migration, and cross-presentation, boosting anti-tumor immunity	cDC1 markers	[103]
	CRC	Notch pathway (mutant)	Tumor-suppressive	CD8^+^ T cells	JAK–STAT, TCR	Mutations enhance chemokine secretion, promoting CD8^+^ T cell infiltration and synergizing with high TMB to activate immunity	Notch pathway mutations	[104, 105]
Stem cell regulation	ESCC	Notch1	Oncogenic	CSCs	TGFβ	Synergizes with the TGFβ–ZEB1 axis to drive the CD44L → CD44H stemness transition	CD44H	[46]
	GC	DLL1/Notch1	Oncogenic	Gastric stem cells (LGR5^+^)	–	H. pylori infection disrupts DLL1^+^ niche signaling to LGR5^+^ stem cells, causing aberrant proliferation	DLL1	[15, 62]
	CRC	Dmt1/Notch	Bidirectional	Intestinal stem cells (ISCs)	Wnt	In an APC-mutant context, Dmt1 isoforms regulate Notch to control the differentiation (suppressive) vs. stemness (oncogenic) balance	Dmt1 isoform ratio	[111, 112]
Inflammation and crosstalk	HCC	Notch1	Oncogenic	Macrophages	c-myc	Activates macrophages via a VCAM1-dependent interaction, promoting lung metastasis	VCAM1	[78]
	PDAC	Jag1/Notch	Bidirectional (late-stage oncogenic)	Macrophages	–	In late stages: establishes a pro-metastatic miR-124-IL-6 feedback loop between tumor cells and macrophages	IL-6, miR-124	[87]
	PDAC	Notch1	Oncogenic	Tumor cells	NF-κB	Cooperates with NF-κB to upregulate Hes1 and suppress the anti-inflammatory protein Pparγ, reinforcing a pro-inflammatory phenotype	Hes1, Pparγ	[92]

### Notch signaling in liver tumors

#### Liver cancer microenvironment and Notch functional switching

In HCC, the Notch signaling pathway undergoes significant microenvironment-dependent functional transitions. Clinical data indicate that high Notch1 expression correlates with accelerated cancer progression, increased lung metastasis, elevated expression of CSC-like gene markers, and reduced overall survival. Specifically, the Notch1–c-myc–VCAM1 signaling axis drives hepatocarcinogenesis by promoting the transformation of liver precursor cells (LPCs) into CSC-like cells, while enhancing spontaneous lung metastasis through VCAM1-dependent macrophage activation [90].

Notably, different Notch ligands exhibit distinct expression patterns during hepatocarcinogenesis. Delta-like 4 (DLL4) is present in both precancerous hepatocytes and HCC cells, while Jag1 is expressed in actin-positive stromal cells [91]. Animal models have shown that hepatocyte-specific knockout of DLL4 eliminates Notch1 signaling and significantly inhibits tumor progression [91]. In tetrachloroethylene-induced HCC, Notch signaling activation is observed, with increased Hes1 expression in tumor areas; the expression levels of DLL4 and Jag1 positively correlate with Hes1. Specifically, DLL4 is primarily expressed in precancerous liver cells and the periphery of HCC, promoting cell proliferation via Notch1 activation. In contrast, Jag1 is expressed in the tumor stroma and activates Notch2 signaling. Functional experiments further confirmed that hepatocyte-specific knockout of DLL4 effectively inhibits Notch1 signaling, reducing HCC incidence and tumor volume. Conversely, Jag1 knockout results in the loss of Notch2 signaling, leading to ectopic DLL4 expression in hepatocytes, which activates the Notch1 pathway aberrantly, increasing HCC incidence and tumor burden. Mechanistically, Notch1 and Notch2 exhibit antagonistic functions: Notch1 promotes hepatocyte proliferation by upregulating Cdk1, while Notch2 inhibits proliferation by inducing Cdkn1/p21 [91–93]. In conclusion, the DLL4/Notch1 signaling pathway promotes HCC progression by driving hepatocyte proliferation, while the Jag1/Notch2 pathway inhibits tumor development by reducing DLL4 expression and Notch1 activation. These dual pathways form a counterbalancing regulatory system that governs the dichotomous roles of Notch signaling in HCC. Moreover, this ligand-dependent regulatory pattern highlights the influence of specific cellular interactions within the TME on Notch signaling outcomes in HCC [91, 94].

#### Mechanisms of Notch3/4 in promoting HCC

Current research indicates that Notch3/4 primarily exerts tumor-promoting effects in the pathogenesis of HCC. The Notch3 pathway operates through the SNORA74A–DCAF13–E2F2–Notch3 axis: SNORA74A is highly expressed in liver CSCs, where it binds DCAF13 to prevent E2F2 ubiquitination and degradation. This, in turn, activates Notch3 transcription and signaling, promoting the self-renewal of liver CSCs and contributing to hepatocarcinogenesis [95]. Consequently, elevated SNORA74A and Notch3 expression serve as indicators of poor prognosis in patients with HCC. Notch4, on the other hand, is specifically required for Nodal expression in aggressive cells and plays a critical role in maintaining cellular growth balance and invasive phenotypes. It is enriched in melanoma cell subpopulations that form vasculogenic mimicry (VM). Inhibition of Notch4 downregulates Nodal expression and impairs VM network formation in invasive melanoma cells, suggesting that Notch4 may be a key regulator of VM formation [96]. Further studies have shown that Notch4 promotes tumor growth by regulating matrix metalloproteinases (MMPs) to influence VM formation, invasion, and progression in HCC [96–98].

### Notch pathway in pancreatic tumors

#### Bidirectional regulation of the Jagged1–Notch axis in pancreatic cancer

The Notch signaling pathway exhibits complex bidirectional regulatory characteristics in pancreatic ductal adenocarcinoma (PDAC). Research has revealed a miR-124-regulated Jagged1–Notch feedback loop that promotes PDAC metastasis through tumor cell–macrophage interactions [89, 99]. The mechanism involves miR-124-mediated suppression of Jagged1/Notch signaling, while activated Notch recruits and activates macrophages through secreted factors. These macrophages then secrete IL-6, which suppresses miR-124 via STAT3 signaling, ultimately promoting EMT and cancer cell invasion [99].

Notably, the absence of Jag1 enhances the development of KrasG12D-induced acinar-to-ductal metaplasia (ADM) and low-grade pancreatic intraepithelial neoplasia (PanIN). However, these pathological changes more frequently progress into benign cystic tumors rather than invasive cancer, suggesting that Jag1 may play a role in restraining early-stage malignant transformation [100]. Therefore, the Jagged1–Notch signaling pathway in PDAC exhibits environment-dependent functional characteristics: it inhibits precancerous transformation at early stages but promotes malignant progression at later stages. The activity of this pathway is influenced by both the cellular environment and tumor development stage, highlighting its potential clinical value as a target for therapeutic intervention.

#### Bidirectional regulation of Notch1 in pancreatic cancer

Notch1 exhibits a dual role in PDAC, contributing both to tumor promotion and regulation of the TME. Its tumor-promoting function manifests in two key aspects: tumor initiation and modulation of the TME. Initially, Notch1 promotes tumorigenesis by maintaining the undifferentiated progenitor cell population in PDAC, particularly in models derived from acinar cells at the early stages of tumor development. For example, in the Kras-driven mouse model, the Notch1 intracellular domain (NICD1) is significantly upregulated in ADM and PanIN lesions [101]. The nuclear localization of NICD1 is associated with the early stages of metaplasia [102, 103]. Secondly, within the TME, Notch1 enhances PDAC progression by regulating the inflammatory response. The NF-κB and Notch signaling pathways act synergistically to upregulate Hes1, which promotes a pro-inflammatory phenotype in tumor cells by inhibiting anti-inflammatory proteins like PPARγ [104].

The anti-cancer function of Notch1 emerges in specific genetic contexts. In the Pdx1-Cre; LSL-Kras^G12D^ mouse model, Notch1 deletion increases the formation and accelerates the progression of PanIN, suggesting that Notch1 may exert a tumor-suppressive effect in malignant tumors derived from embryonic pancreatic progenitor cells [105]. Avila et al. proposed that Notch1 limits excessive progenitor cell proliferation by maintaining differentiation, and its deletion leads to the accumulation of undifferentiated cells [106]. Therefore, Notch1’s role in PDAC depends on both the cell of origin (mature acinar cells vs. embryonic precursor cells) and the tumor stage (initiation vs. progression): it typically promotes carcinogenesis in PDAC derived from adult acinar cells, while exhibiting anti-cancer effects in tumors derived from embryonic precursor cells or under specific genetic conditions. This complexity highlights the need for precise, context-dependent intervention when targeting Notch1 in cancer therapy.

#### Bidirectional regulation of Notch in pancreatic neuroendocrine tumors

In pancreatic neuroendocrine tumors (PNETs), Notch signaling also exhibits dual functionality. PNETs are a heterogeneous group of pancreatic neoplasms displaying neuroendocrine differentiation, constituting the second most common form of pancreatic cancer after PDAC. In its oncogenic capacity, Notch pathway activity regulates the transcription and nuclear positioning of INSM1 via p53 activation. INSM1 is a key transcriptional regulator that controls proliferation in PNET cells. In the presence of MEN1 deficiency and a wild-type p53 status, increased nuclear accumulation of INSM1 promotes cellular proliferation. Conversely, inhibiting Notch reduces the nuclear translocation of INSM1, thus inhibiting proliferation in PanNET cells. The regulatory effect of Notch on INSM1 and cell proliferation is dependent on p53 status; p53 mutations disrupt this mechanism [107]. Regarding its tumor-suppressive role in PNETs, histopathological analysis has shown reduced Notch1 expression in malignant PNETs and those of indeterminate behavior, whereas benign tumors exhibit some degree of preserved expression [108]. Additionally, in mixed neuroendocrine–non-neuroendocrine neoplasms (MiNENs), Notch1 and Hes1 expression are significantly reduced or completely absent in neuroendocrine regions, although these markers remain present in the glandular tumor compartments [109]. These findings suggest that Notch may function as a tumor suppressor in certain pancreatic tumors.

### Research progress on the Notch pathway in colorectal cancer

#### Tumor-promoting role of Notch in colorectal cancer

**Fig. 3:**
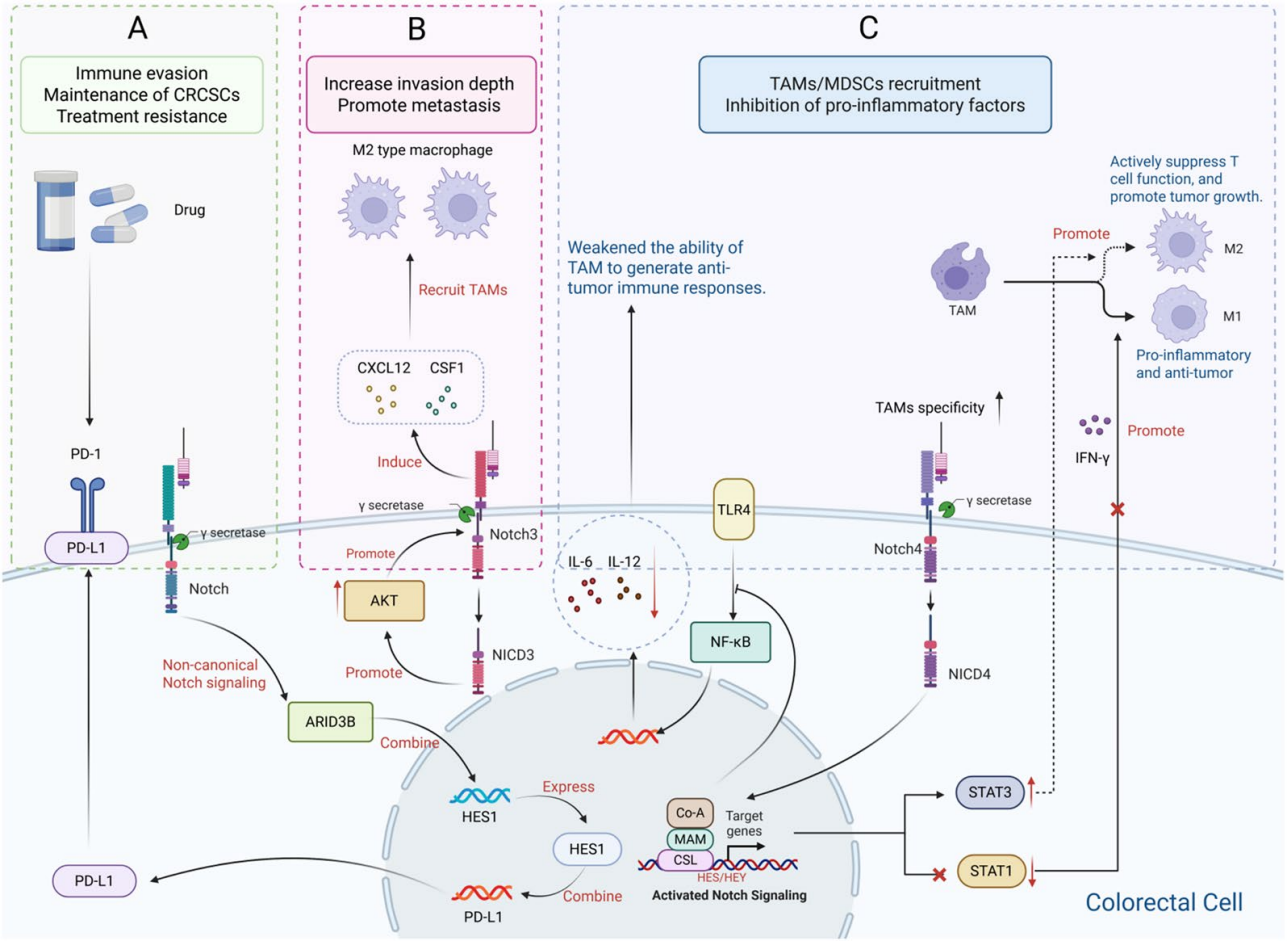
The Notch signaling pathway promotes the progression of CRC by shaping an immunosuppressive microenvironment. **A** The non-canonical Notch pathway mediates immune evasion and therapy resistance by upregulating PD-L1 expression on colorectal cancer stem cells (CRCSCs). **B** Activation of Notch3 recruits macrophages, promotes tumor growth and liver metastasis, and its high expression is associated with poor prognosis. **C** Upregulation of Notch4 in TAMs enhances their immunosuppressive phenotype by suppressing the release of pro-inflammatory cytokines through the remodeling of key signaling pathways such as STAT1, STAT3, and NF-κB, while also integrating multiple pathways to maintain inflammatory balance. (Created by biorender.com)

The Notch pathway facilitates colorectal cancer (CRC) progression by shaping an immunosuppressive microenvironment. Aberrant activation of Notch signaling promotes immune escape by modulating the function and balance of myeloid-derived suppressor cells (MDSCs) and TAMs. For example, upregulation of Notch4 in TAMs suppresses the release of pro-inflammatory cytokines (such as IL-6 and IL-12) and enhances an immunosuppressive phenotype by inhibiting STAT1 phosphorylation and transcriptional activity, increasing STAT3 activation, and disrupting TLR4–NF-κB signaling. Moreover, Notch4 integrates the Notch, IFN-γ, and TLR4 pathways through regulation of HES1 and canonical Notch signaling, maintaining inflammatory balance in the TME (Fig. 3. C) [110].

In tumor progression and metastasis, Notch1 overexpression correlates with advanced pathological features in CRC, including depth of invasion and lymph node metastasis [111]. Notch3 activation contributes to the formation of an immunosuppressive microenvironment, facilitating tumor growth and liver metastasis by upregulating cytokines such as CSF1 and CXCL12, which recruit macrophages and increase the infiltration of both macrophages and MDSCs into the CRC TME. Studies suggest that AKT-dependent Notch3 activation is a key driver of invasion and metastasis in CMS4-type CRC (Fig. 3. B). Mouse model studies have shown that inhibiting Notch3 reduces tumor progression. Consistently, high Notch3 expression in human CMS4 patients is linked to poor prognosis [112, 113]. Notably, through the non-canonical Notch pathway (NICD-independent), Notch signaling upregulates PD-L1 via the ARID3B–HES1 axis, sustaining the immune evasion capacity of CRCSCs and contributing to therapy resistance (Fig. 3. A) [114].

#### Anti-tumor role of Notch in colorectal cancer

In inflammation-associated CRC, Notch2 signaling regulates dendritic cells (DCs) to suppress the development of inflammation-associated CRC through anti-tumor immunity. Wang et al. demonstrated in murine models and human CRC sample analyses that Notch2 signaling deficiency impairs the differentiation, migration, and cross-presentation capabilities of conventional DCs (cDC1), thereby promoting inflammation-driven carcinogenesis. Conversely, Notch-activated DCs effectively inhibit tumor progression. In human CRC specimens, cDC1 infiltration positively correlates with Notch2 signaling activity, while GMDS-mutant CRC shows reduced expression of cDC1 signature genes and a poorer prognosis [115]. These findings emphasize the pivotal role of the microbiota–immune–Notch axis in tumorigenesis. Mutations in the Notch pathway (Notch-MT) enhance chemokine secretion within the TME, promote CD8^+^ T cell infiltration, and strengthen anti-tumor immune responses, particularly in microsatellite instability-high (MSI-H) CRC. The Notch-MT group demonstrates heightened immunogenicity, characterized by increased infiltration of immune cells such as M1 macrophages, CD8^+^ T cells, neutrophils, and activated NK cells, as well as a significantly elevated TMB, neoantigen load (NAL), and mutation counts in DNA damage repair (DDR) pathways. Concurrently, this group shows upregulation of immune checkpoint genes (e.g., CD274, PDCD1), enrichment in immune-related pathways such as JAK–STAT and T-cell receptor signaling, and downregulation of pathways like Wnt. These mutations are associated with improved prognosis due to enhanced immune infiltration and immunogenicity (e.g., high TMB and NAL). Clinical studies have demonstrated significantly prolonged overall survival in patients with Notch-MT CRC treated with ICIs [116, 117]. Regarding cellular differentiation, the Notch pathway also plays a role in suppressing tumor growth. By regulating cell cycle-related genes (e.g., p27), Notch signaling controls the proliferation rate of transit-amplifying cells, maintaining a dynamic balance between differentiation and shedding [118]. Loss of Notch function may lead to unrestrained inhibition of p27, resulting in excessive proliferation, disrupted differentiation, abnormal expansion of undifferentiated cells, and an increased tumorigenic risk [119, 120].

In the context of CRC, the Notch pathway primarily promotes oncogenic activity by remodeling the immune microenvironment and facilitating metastatic processes, thus accelerating disease progression. Paradoxically, genetic alterations or suppression of Notch signaling can yield anti-tumor outcomes by stimulating immune responses against cancer. This dual functionality suggests that therapeutic strategies targeting Notch must be carefully tailored based on individual molecular profiles and immunological conditions, allowing for more effective and personalized interventions that ultimately improve prognostic outcomes.

#### Mechanisms of intestinal stem cell homeostasis regulation

Mutations in the adenomatous polyposis coli (APC) gene are a critical event in colorectal carcinogenesis, present in over 80% of sporadic CRC cases [121]. The Notch signaling pathway plays a key role in regulating intestinal stem cell (ISC) fate. APC mutations can arise in ISCs through conventional self-renewal or by dedifferentiation of their progeny [122]. In APC-deficient intestinal organoids, the expression of the iron transporter Dmt1 isoform shows a close correlation with Notch and Wnt signaling, playing dual roles in tumorigenesis [123, 124]. Dmt1 is essential for regulating Notch signaling; its deficiency suppresses both ligand-dependent and ligand-independent Notch activation, along with disrupted endolysosomal trafficking and accumulation of reactive oxygen species (ROS). This regulation is isoform-specific: depletion of Dmt1-ire reduces Notch target gene expression, promoting differentiation of myocytes, neurons, and intestinal epithelial cells while inhibiting tumorigenesis. In contrast, Dmt1 + ire deficiency enhances Notch signaling, maintaining stem/progenitor cell properties and facilitating tumor development. In intestinal organoid models, this effect is distinctly observed: Dmt1−ire knockdown induces mature crypt formation, while Dmt1 + ire knockdown promotes undifferentiated spheroid formation, a process associated with Wnt/Notch signaling synergy in APC-deficient models. In CRC cells, Dmt1-ire deletion mimics γ-secretase inhibitor (GSI) effects by suppressing Notch signaling, promoting goblet cell differentiation, reducing stem cell marker expression, and inhibiting colorectal carcinogenesis. Clinical sample analysis supports the pathological relevance, showing a significant correlation between Dmt1 isoform expression ratios and Notch target genes (e.g., Hes4), suggesting that Dmt1-mediated Notch regulation contributes to clinically relevant pathophysiological processes [124]. These findings highlight the central role of Dmt1 isoforms in cellular fate determination, providing novel mechanistic insights into Notch-dysregulated CRC and identifying potential therapeutic targets for drug development.

### Current therapeutic advances

#### Targeting Notch signaling pathway in digestive system cancers: therapeutic challenges

As previously discussed, the Notch signaling cascade is a fundamental mechanism for intercellular communication. Through specific ligand–receptor interactions at cellular interfaces, this pathway precisely regulates downstream gene expression, exerting critical influence over essential biological processes such as cellular differentiation, proliferation, programmed cell death, and stem cell maintenance. However, in many digestive tract malignancies, dysregulated activation of Notch signaling often correlates with increased tumor invasiveness, metastatic potential, and therapeutic resistance [119]. For example, Notch1 and Notch3 are frequently activated in gastric cancer and CRC, further promoting tumor growth and malignant transformation. Notably, Notch signaling can both promote and inhibit tumorigenesis, depending on the context, which adds complexity to its role in cancer biology. This dual functionality poses significant challenges for nonspecific inhibitors, such as GSIs, which target the entire Notch pathway and are associated with wide-ranging toxicities (e.g., gastrointestinal toxicity), severely limiting their clinical application. Thus, the development of drugs that selectively target specific Notch receptor subtypes (Notch1–4) to achieve precise inhibition is key to overcoming this clinical barrier. Current research on the targeted inhibition of Notch signaling in digestive system cancers—such as PDAC, HCC, and ESCC—focuses on further elucidating the synergistic mechanisms between pathway activation and downstream regulatory factors to develop more effective combination treatment strategies. For instance, studies have shown that IL-17 and the Notch signaling pathway work synergistically to promote the progression of PDAC, suggesting that combined inhibition of both may offer a novel therapeutic direction for PDAC treatment [125]. In HCC, miR-3163 inhibits Notch signaling activation by targeting ADAM-17, thereby enhancing the sensitivity of HCC cells to sorafenib and other existing targeted therapies [126]. Furthermore, MAML1, a key component of the Notch transcriptional complex, plays a central role in maintaining ESCC stem cells (CSCs); as such, targeting MAML1 is emerging as a promising strategy to overcome CSC resistance in ESCC [127]. Although these studies are primarily based on in vitro and animal models and lack large-scale clinical validation, they collectively highlight the substantial potential of the Notch pathway and its associated molecules as therapeutic targets for digestive system cancers. These findings provide a clear direction for the future development of more precise and effective targeted therapies.

#### Microenvironment remodeling combined therapy strategies

Current research on microenvironment-based therapies focuses on deeply understanding the specific regulatory mechanisms of the Notch signaling pathway within the TME of various digestive system cancers, and its interactions with other TME components such as cancer-associated fibroblasts (CAFs), immune cells, and the extracellular matrix (ECM) [128–131]. Researchers are actively exploring new treatment strategies for the Notch signaling pathway, including the use of small-molecule inhibitors and immunotherapy. These strategies emphasize the importance of combination therapy to improve efficacy and reduce drug resistance [75]. However, the existing studies have notable limitations, such as an incomplete understanding of the Notch signaling pathway mechanisms in specific tumor types [132]. Future research should focus on the in-depth elucidation of Notch signaling mechanisms in the TME, the development of more selective targeted therapies, and the optimization of combination therapies, all aimed at providing more effective treatments for patients with digestive system cancers [133]. From a translational perspective, these mechanistic insights suggest several concrete paths toward clinical application. Early-phase trials in ESCC, GC, HCC, PDAC, and CRC could prospectively stratify patients by Notch receptor/ligand expression, mutation status, or Notch-related immune signatures, and then test subtype-selective agents (e.g., anti-Notch1/3 antibodies, DLL4/Jag1 blockers, or γ-secretase modulators deployed in biomarker-defined cohorts), either as monotherapy or in rational combinations with chemotherapy, anti‑angiogenic agents, or immune checkpoint inhibitors. Embedding correlative endpoints such as dynamic changes in cancer stem cell markers, vascular density, and effector T-cell infiltration would directly connect Notch pathway modulation to clinical benefit and guide refinement of patient selection and regimen design in subsequent phase II/III studies.

## Conclusions and prospects

The Notch signaling pathway exhibits significant tissue-specific regulatory characteristics across different gastrointestinal tumors, and understanding the molecular mechanisms driving these differences remains a key area of research. As mentioned earlier, Notch3 is continuously upregulated in gastric cancer and is associated with malignant progression [78, 79], but it may play a tumor-suppressive role in other tumor types. This tissue specificity may arise from differential expression profiles of receptor subtypes, integration of microenvironmental signals, or regulation by epigenetic modifications. Notably, significant variations in Notch signaling activity have been observed even between different anatomical regions of the same organ, such as the gastric antrum and gastric body [21, 88]. This presents a major challenge for precision medicine. The dual role of the Notch signaling pathway in digestive system cancers—acting both as an oncogene and a tumor suppressor gene—exhibits complex characteristics. In esophageal cancer, Notch1 mutations may inhibit tumor progression, whereas in gastric cancer, activation of Notch1 and Notch2 promotes tumor proliferation and metastasis. Pancreatic cancer exhibits bidirectional regulation of Jagged1–Notch signaling, acting as a tumor suppressor in early stages but promoting metastasis in advanced stages. In colorectal cancer, Notch signaling drives tumor progression through immune evasion, with the function of different Notch receptors potentially dependent on specific immune microenvironments. These findings suggest that precision therapy targeting Notch signaling requires consideration of specific receptor subtypes combined with immunomodulatory strategies. Future research should focus on exploring the role of Notch signaling within the tumor microenvironment, leveraging single-cell technologies to provide new directions for precision targeted therapy. This approach aims to enhance cancer treatment efficacy and overcome drug resistance challenges.

## Data Availability

Not applicable.

## Abbreviations

GSIs: γ-Secretase inhibitors
ASDR: Age-standardized mortality rates
EMT: Epithelial–mesenchymal transition
LUSC: Lung squamous cell carcinoma
NECD: Notch extracellular domain
NTM: Notch transmembrane domain
NICD: Notch intracellular domain
EGF: Epidermal growth factor
NRR: Negative regulatory region
bHLH: Basic helix-loop-helix
CSL: CBF1/suppressor of hairless/Lag1
RBPJ: Recombination signal binding protein for immunoglobulin kappa J region
ESCC: Esophageal squamous cell carcinoma
TGF-β: Transforming growth factor-β
ZEB1: Zinc Finger E-Box Binding Homeobox 1
STAT3: Signal transducer and activator of transcription 3
USP5: Ubiquitin-specific protease 5
VEGF: Vascular endothelial growth factor
TAMs: Tumor-associated macrophages
CRT: Chemoradiotherapy
NRF2: Nuclear factor erythroid 2-related factor 2
DLL1: Delta like canonical notch ligand 1
LGR5: Leucine rich repeat containing G protein-coupled receptor 5
CARM1: Coactivator associated arginine methyltransferase 1
N2ICD: Notch2 intracellular domain
MAML1: Mastermind like transcriptional coactivator 1
Akt: AKT serine/threonine kinase
mTOR: Mechanistic target of rapamycin
ICI: Immune checkpoint inhibitor
TMB: Tumor mutation burden
PD-1: Programmed cell death protein 1
PD-L1: Programmed cell death ligand 1
CTLA4: Cytotoxic T-lymphocyte associated protein 4
HCC: Hepatocellular carcinoma
LPCs: Liver precursor cells
DLL4: Delta like canonical notch ligand 4
Jag1: Jagged canonical notch ligand 1
CSCs: Cancer stem cells
VM: Vasculogenic mimicry
MMPs: Matrix metalloproteinases
PDAC: Pancreatic ductal adenocarcinoma
ADM: Acinar-to-ductal metaplasia
PanIN: Pancreatic intraepithelial neoplasia
NF-κB: Nuclear factor kappa B
PPARγ: Peroxisome proliferator activated receptor gamma
PNETs: Pancreatic neuroendocrine tumors
INSM1: Insulinoma associated 1
MEN1: Multiple endocrine neoplasia type 1
MiNENs: Mixed neuroendocrine–non-neuroendocrine neoplasms
CRC: Colorectal cancer
MDSCs: Myeloid-derived suppressor Cells
TLR4: Toll-like receptor 4
IFN-γ: Interferon gamma
DCs: Dendritic cells
cDC1: Conventional type 1 dendritic cells
MSI-H: Microsatellite instability-high
NAL: Neoantigen load
DDR: DNA damage repair
APC: Adenomatous polyposis coli
ISCs: Intestinal stem cells
Dmt1: Divalent metal transporter 1
ROS: Reactive oxygen species
CAFs: Cancer-associated fibroblasts
ECM: Extracellular matrix
